# Aquatic Plants in Ponds at the Brdo Estate (Slovenia) Show Changes in 20 Years

**DOI:** 10.3390/plants13172439

**Published:** 2024-08-31

**Authors:** Mateja Germ, Monika Bajc Tomšič, Igor Zelnik, Nik Ojdanič, Aleksandra Golob

**Affiliations:** Department of Biology, Biotechnical Faculty, University of Ljubljana, Jamnikarjeva 101, 1000 Ljubljana, Slovenia; monika.bajc@gmail.com (M.B.T.); igor.zelnik@bf.uni-lj.si (I.Z.); nik.ojdanic@bf.uni-lj.si (N.O.); aleksandra.golob@bf.uni-lj.si (A.G.)

**Keywords:** submerged aquatic plants, emergent plants, macrophytes, ponds, ecological types

## Abstract

Ponds are important habitats for aquatic plants and other biota, particularly in regions where the quality of aquatic ecosystems is deteriorating or even disappearing. Ponds provide refuge for many species and serve as foraging places for others. The ponds studied are located in the Brdo Estate and are under special protection to maintain their educational and other ecosystem services. This study examined the temporal differences (20 years) of the plant communities in eleven ponds concerning eutrophication and/or other human pressures. Various measurements were taken between the two surveys to improve the quality of inflowing water. The selected ponds’ physical and chemical parameters, water depth, and transparency were measured. According to our results, water transparency and temperature significantly shaped the structure of the plant community and significantly influenced the presence and abundance of aquatic plants. The changes were reflected in the disappearance of four species of the genus *Potamogeton*, namely *P. filiformis*, *P. lucens*, *P. pectinatus*, and *P. trichoides*, which were recorded in 2001 but not in 2021. Secondly, the average number of plant species in the ponds has slightly increased in 20 years, mainly due to emergent plants. The construction of wastewater treatment plants in the catchment area prevented the eutrophication processes.

## 1. Introduction

The changes in the presence and abundance of aquatic plant species indicate the changes in ecosystems [1], so they can be used to assess the ecological status of lakes [2,3]. Aquatic plants have an essential structuring role in shallow standing waters [4]. They are important to fish and other wildlife communities in ponds, providing cover, nesting sites, and food. Aquatic plants or macrophytes significantly increase habitat complexity for other groups of aquatic organisms [5,6,7], resulting in a greater number of microhabitats per unit area and, consequently, greater biodiversity. Higher complexity can also be achieved by a higher complexity of functional types of plants, which have different roles in aquatic ecosystems. For instance, submerged aquatic plants are important in enabling good water quality in shallow standing waters [8], with intensive oxygen supply to the water.

Ponds are small, lentic water bodies that do not exceed 1 hectare (0.01 km^2^) in surface area but account for 99% of all standing water bodies and 31% of all standing water bodies in the world in terms of surface area [9]. Some researchers limit the surface area of a pond to up to 2 hectares [10]. Despite their high numbers, research on these water bodies is less common than research on larger lakes. Ponds and small lakes provide many important ecosystem services, including water retention and nutrient cycling [11]. These aquatic habitats are also important for biodiversity [12], and when degraded, the biota is unified at local and regional scales [13]. Ponds also play an essential role in the carbon cycle [14] and can be an additional tool to mitigate C emissions [15]. Small and artificial lakes are very important for maintaining the diversity of submerged and emergent aquatic plant species compared to large and natural lakes [16]. Small standing waters have broad ecotones, sometimes even corresponding to their entire surface area, which increases structural heterogeneity, enabling extremely high biodiversity and metabolic rates [17]. For example, it has been reported that the diversity of aquatic plants in the relatively small pond in Tohoku District, Japan, showed remarkable richness compared to natural lakes [18].

Eutrophication is a natural process caused by a natural nutrient source, such as a deciduous forest next to a pond [10], which contributes to increased concentrations of nitrogen and phosphorus [19]. On the other hand, Akasaka et al. [20] reported that urban areas that dominate the catchment area within 500 m of a pond negatively influence the species diversity of aquatic plants, especially the diversity of submerged and natant species. In general, eutrophication of waterbodies refers to the increase in trophic status due to the enrichment with nutrients, especially nitrogen and phosphorus, with increasing discharge from agricultural land, industry, and municipal wastewater [21]. Körner [22] reported that decades of excessive nutrient loading in many shallow lakes have led to eutrophication and a strong increase in water turbidity. Long-term eutrophication processes have led to a decline in the species richness of aquatic plants in shallow standing waters [23,24]. Such habitats have become less suitable for the growth of submerged macrophytes, resulting in the loss of many submerged species [22,23,25]. Zhang et al. [26] reported that the loss of submerged macrophytes accounts for almost two-thirds of extinct aquatic plants.

The processes of natural succession of shallow water bodies are usually facilitated by eutrophication [27]. The intensive primary production of emergent macrophytes and phytoplankton in responsible for large amounts of organic matter that accumulates in the littoral zone and enables terrestrialization of these waters [28,29]. With the establishment of species-poor stands of successful, tall-growing emergent macrophytes or woody species such as willows, these habitats are unified and lose their diversity of microhabitats and, consequently, aquatic plant species [30].

The aim of the study was to determine the temporal differences in the occurrence and abundance of aquatic plant species and the structure of the communities they form in eleven ponds on the Brdo Estate. Although the studied ponds are relatively shallow and subject to rapid eutrophication and succession processes, we hypothesized that this would not be reflected in the macrophyte community structure. We hypothesized that the species richness and diversity of macrophytes would increase, and the proportion of nitrophilous species would decrease over 20 years, as the quality of the inflowing water improved between the two surveys due to the suitable measurements. Our second hypothesis assumed that the proportions of plant ecological types would change as successional processes took place over 20 years.

## 2. Results

### 2.1. Presence and Abundance of Macrophyte Taxa in Years 2001 and 2021

Table 1 shows the presence and abundance of all 52 macrophyte taxa found in the Brdo Estate ponds during the 2001 and 2021 surveys.

The presence of submerged and natant macrophytes in the surveyed ponds changed from 2001 to 2021 (Table 1). In twenty years, the total number of taxa increased from 41 to 43. The taxa thriving in more than half of the ponds in both years are the following: *Chara* sp., *Caltha palustris*, *Galium palustre*, *Lycopus europaeus*, *Mentha aquatica*, *Solanum dulcamara*, and *Typha latifolia*.

In 2021, seventeen taxa of macrophytes were thriving in a smaller number of ponds, twenty-nine in a larger number of ponds, and eight in an equal number of ponds compared to the 2001 results.

**Figure 1:**
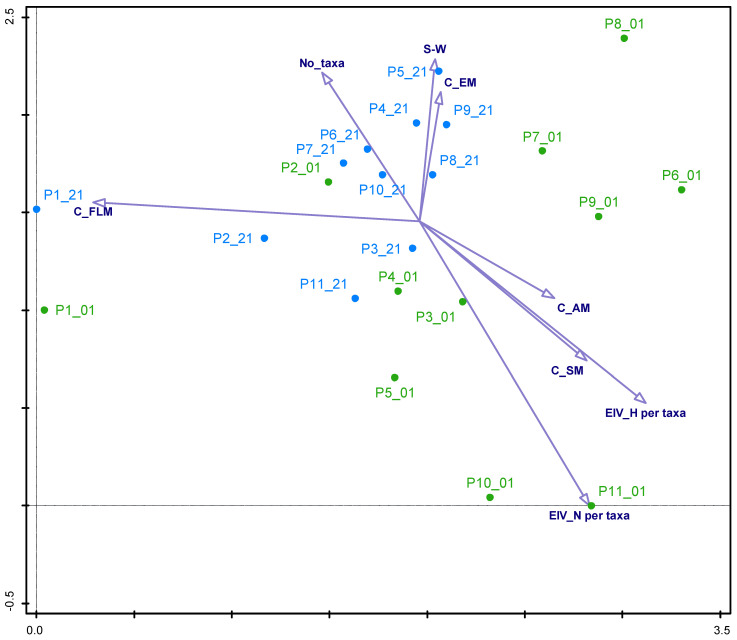
Ordination diagram of macrophyte communities, based on DCA with passively projected gradients); EIV_H per taxa—total Ellenberg’s humidity indicator values (EIV_N); EIV_N per taxa—total Ellenberg’s nutrient indicator values (EIV_N); No_taxa—number of taxa; C_SM—coverage with submerged macrophytes; C_AM—coverage with amphiphytes; C_FLM—coverage with floating-leaved macrophytes; C_EM—coverage with helophytes S-W—Shannon–Wiener diversity index; blue color—survey in 2021; green color—survey in 2001.

We observed a decrease in the number of submerged at the expense of an increase in the number of emergent macrophytes (Table 1). However, the differences in the cover of submerged and emergent macrophytes were not statistically significant (*p* = 0.085, *p* = 0.278, respectively (Supplementary Materials Figures S1 and S2). A more evident shift in macrophyte community structure over 20 years can be seen in Figure 1, and it has changed along the hydrological gradient based on EIV for humidity.

**Figure 2:**
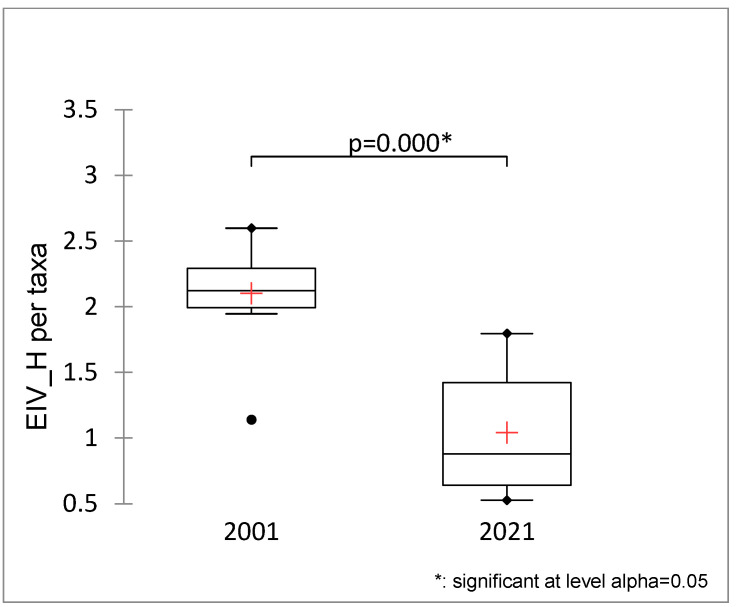
Total Ellenberg indicator values for humidity (EIV_H) for Brdo ponds in 2001 and 2021 (Mann–Whitney test). +—average value, dot—outliers.

This change within a 20-year period towards more emergent vegetation is reflected in the significant decrease in total Ellenberg indicator values for humidity between 2001 and 2021 (Figure 2).

**Figure 3:**
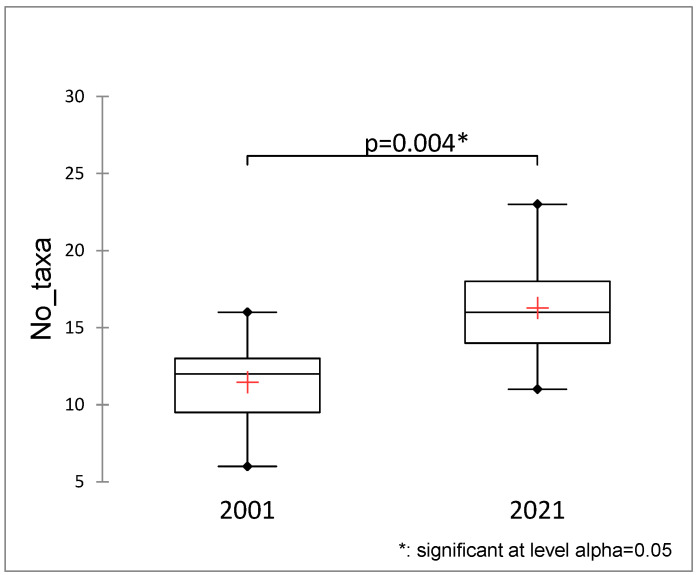
Average numbers of species in Brdo ponds in 2001 and 2021 (Mann–Whitney test). +—average value, dot—outliers.

The average number of species found in the ponds increased slightly between 2001 and 2021 (Figure 3). However, there were no significant differences in the Shannon–Wiener diversity index between the years.

**Figure 4:**
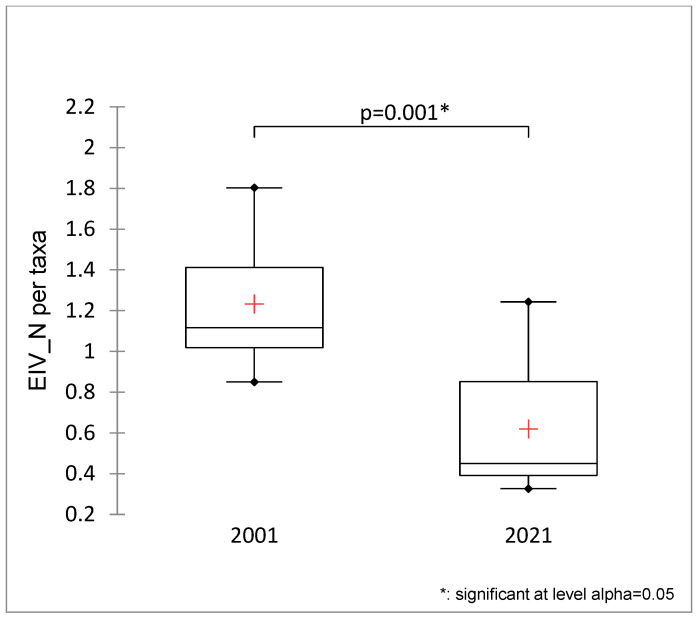
Total Ellenberg’s nutrient indicator values (EIV_N) for Brdo ponds for the years 2001 and 2021 (Mann–Whitney test). +—average value, dot—outliers.

The Ellenberg indicator values for nutrients (EIV_N) was significantly lower in 2021 than in 2001 (Figure 4).

### 2.2. Effect of Physical and Chemical Properties on the Distribution of Macrophytes in Ponds in 2021

The depth of the ponds investigated in 2021 ranged from 1 m (pond 1) to 4.5 m (pond 11), whereas the Secchi depth ranged from 0.8 to 2.95 m (Table 2).

**Figure 5:**
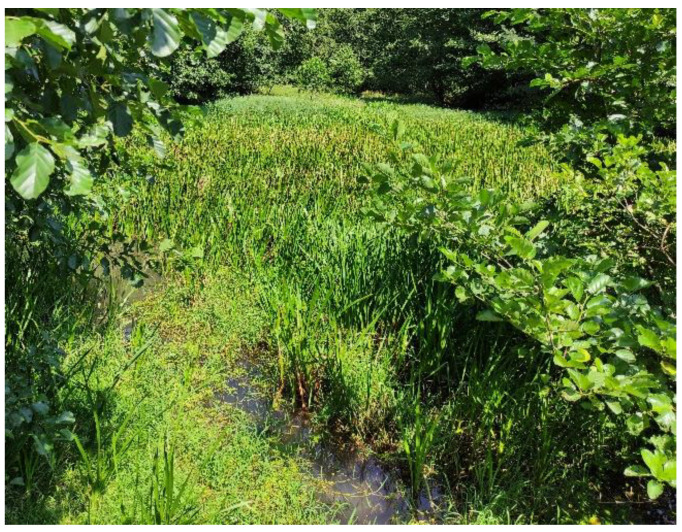
Pond 9 is densely overgrown with emergent macrophytes (helophytes).

The temperature in the ponds ranged from 11.5 to 19.9 °C (Table 2). The lowest temperature measured was in pond 9 (11.5 °C) and the highest in pond 6 (19.9 °C). Pond 9 is completely overgrown with various emergent macrophytes (Figure 5).

The electrical conductivity in the ponds ranges from 364 (P6) to 4l7 µS/cm (P1) (Table 2).

**Figure 6:**
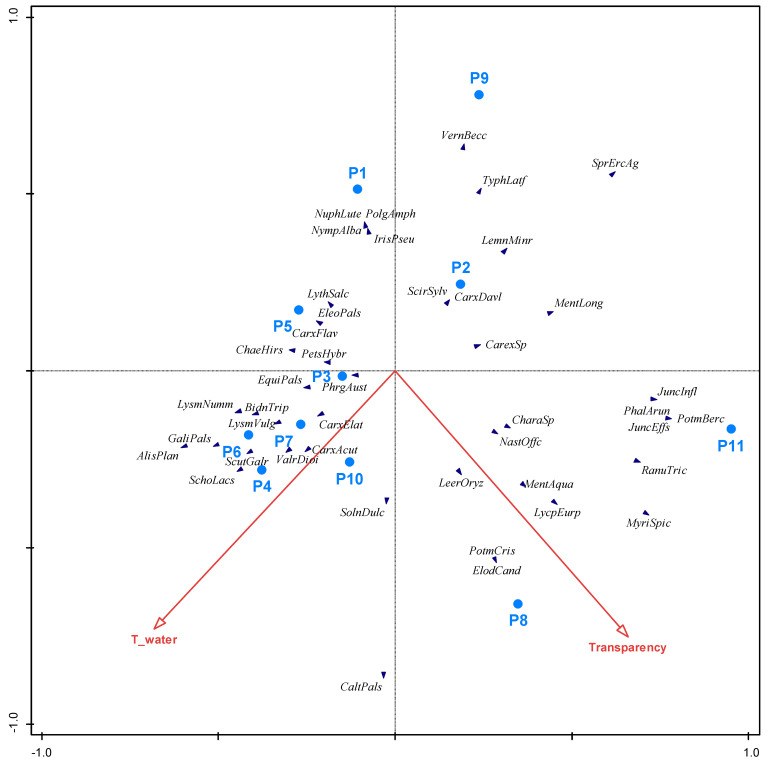
The influence of physical and chemical factors on the occurrence and abundance of all listed macrophyte taxa in 2021; Arrows represent the direction of increasing transparency and temperature of the water, respectively, T_water—temperature of water; AlisPlan—*Alisma plantago-aquatica*, BidnTrip—*Bidens tripartita*, CaltPals—*Caltha palustris*, CarxAcut—*Carex acuta*, CarxElat—*Carex elata*, CarxDavl—*Carex davalliana*, CarxFlav—*Carex flava*, CarexSp—*Carex* sp., ChaeHirs—*Chaerophyllum hirsutum*, CharaSp—*Chara* sp., ElodCand—*Elodea canadensis*, EleoPals—*Eleocharis palustris*, EquiPals—*Equisetum palustre*, GaliPals—*Galium palustre*, IrisPseu—*Iris pseudacorus*, JuncEffs—*Juncus effusus*, JuncInfl—*Juncus inflexus*, LeerOryz—*Leersia oryzoides*, LemnMinr—*Lemna minor*, LycpEurp—*Lycopus europaeus*, LysmNumm—*Lysimachia nummularia*, LysmVulg—*Lysimachia vulgaris*, LythSalc—*Lythrum salicaria*, NastOffc—*Nasturtium officinale*, NuphLute—*Nuphar luteum*, NympAlba—*Nymphaea alba*, MentAqua—*Mentha aquatica*, MentLong—*Mentha longifolia*, MyriSpic—*Myriophyllum spicatum*, PetsHybr—*Petasites hybridus*, PhalArun—*Phalaris arundinacea*, PhrgAust—*Phragmites australis*, PolgAmph—*Polygonum amphibium*, PotmBerc—*Potamogeton berchtoldii*, PotmCris—*Potamogeton crispus*, RanuTric—*Ranunculus trichophyllus*, ScirSylv—*Scirpus sylvaticus*, ScutGalr—*Scutellaria galericulata*, SprErcAg—*Sparganium erectum* agg., SchoLacs—*Schoenoplectus lacustris*, SolnDulc—*Solanum dulcamara*, TyphLatf—*Typha latifolia*, ValrDioi—*Valeriana dioica*, and VernBecc—*Veronica beccabunga*.

The RDA analysis, in which we included all listed macrophyte taxa and measured physical and chemical factors, showed that water temperature and transparency (Secchi depth) had a statistically significant effect (*p* = 0.004, Monte Carlo permutation test) on the presence and abundance of macrophytes in the ponds (Figure 6).

**Figure 7:**
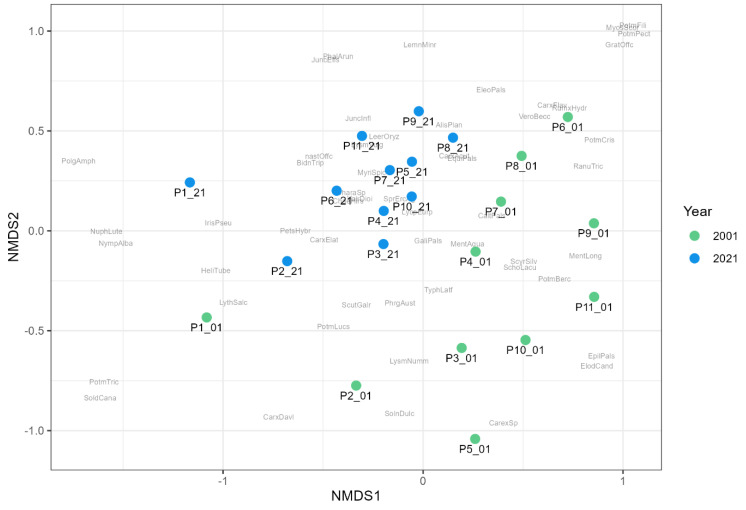
The differences between the macrophyte communities based on taxonomic composition according to NMDS (stress = 0.1991). Green circles—ponds surveyed in 2001; blue circles—ponds surveyed in 2021. AlisPlan—*Alisma plantago-aquatica*, BidnTrip—*Bidens tripartita*, CaltPals—*Caltha palustris*, CarxAcut—*Carex acuta*, CarxElat—*Carex elata*, CarxDavl—*Carex davalliana*, CarxFlav—*Carex flava*, CarexSp—*Carex* sp., ChaeHirs—*Chaerophyllum hirsutum*, CharaSp—*Chara* sp., ElodCand—*Elodea canadensis*, EleoPals—*Eleocharis palustris*, EquiPals—*Equisetum palustre*, GaliPals—*Galium palustre*, IrisPseu—*Iris pseudacorus*, JuncEffs—*Juncus effusus*, JuncInfl—*Juncus inflexus*, LeerOryz—*Leersia oryzoides*, LemnMinr—*Lemna minor*, LycpEurp—*Lycopus europaeus*, LysmNumm—*Lysimachia nummularia*, LysmVulg—*Lysimachia vulgaris*, LythSalc—*Lythrum salicaria*, NastOffc—*Nasturtium officinale*, NuphLute—*Nuphar luteum*, NympAlba—*Nymphaea alba*, MentAqua—*Mentha aquatica*, MentLong—*Mentha longifolia*, MyriSpic—*Myriophyllum spicatum*, PetsHybr—*Petasites hybridus*, PhalArun—*Phalaris arundinacea*, PhalArun—*Phalaris arundinacea*, PhrgAust—*Phragmites australis*, PolgAmph—*Polygonum amphibium*, PotmBerc—*Potamogeton berchtoldii*, PotmCris—*Potamogeton crispus*, RanuTric—*Ranunculus trichophyllus*, ScirSylv—*Scirpus sylvaticus*, ScutGalr—*Scutellaria galericulata* LemMinr_—Lemna Minor, and PolgAmph—*Polygonum amphibium*.

**Figure 8:**
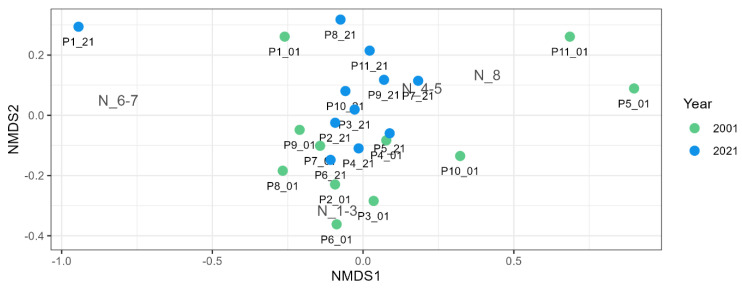
The differences between the macrophyte communities based on the preference of macrophytes for nutrients according to NMDS (stress = 0.1040). Green circles—ponds surveyed in 2001; blue circles—ponds surveyed in 2021. N_1-3—low nutrient requirements; N_4-5—medium nutrient requirements; N_6-7—high nutrient requirements; N_8—very high nutrient requirements.

Among the factors measured, only transparency, measured as Secchi depth, and water temperature significantly affected the presence and abundance of macrophytes. A series of NMDS analyses revealed a unification of macrophyte assemblages within 20 years when the taxa matrix was used (Figure 7). According to ANOSIM analysis, there was mild but significant difference in plant community (R = 0.3833, *p* = 0.0001) between the years 2001 and 2021. Figure 8 also showed a shift in preference for nutrients according to EIV_N (*p* = 0.0214, ANOSIM); however, R value was very low at 0.1452, showing low dissimilarity between both years.

## 3. Discussion

### 3.1. Presence and Abundance of Macrophyte Taxa in Years 2001 and 2021

Ponds are important water bodies that mitigate the decline in aquatic biodiversity, and when degraded, the unification of biota occurs at local and regional scales [13,31]. Our results show that the average number of species found in the ponds increased significantly (*p* = 0.004) from 2001 to 2021. Still, no significant differences existed between years in the Shannon–Wiener diversity index. The increase in the number of taxa in 2021 was almost entirely due to the increase in the number of emergent macrophytes. This shift towards less-wet sites is consistent with the proportion of functional types showing a change towards more emergent vegetation (Figure 1), resulting in a unification of community structure (Figure 1). However, the unification was supposed to be much more intense, as it is the consequence of successional and terrestrialization processes [29]. We found that the presence and abundance of submerged macrophytes have declined in most ponds on Brdo Estate, where these species thrived 20 years ago. One of the reasons might also be the consequences of climate change, especially the increase in the frequency of heavy rain events during the year. Submerged plants are more vulnerable to water level fluctuation than emergent plants, and they cannot survive substantial changes in water availability. For example, in Lake Liangzi, eleven submerged plant species were detected in 2009, but after the flood, their number dropped to five two years later [32]. Alahuhta et al. [33] stated that increased rainfall and subsequent run-off of nutrients from the terrestrial environment to water will stimulate the growth of emergent macrophytes. Lind et al. [34] recently also reported that due to effects of climate change, the abundance and distribution of submerged macrophytes will probably decrease, while the growth of emergent and floating species will increase. The presence and abundance of submerged macrophytes have declined in most ponds on Brdo Estate, where these species thrived 20 years ago. A decline in the abundance of submerged macrophytes was observed in northeast Germany in the 1940s [22]. Körner [22] states that in many lakes, especially the shallowest ones, submerged macrophytes no longer thrive after four decades. An increase in emergent vegetation and the shrinking of open water surface are typical phenomena in the succession of ponds [35].

It is known that nutrient enrichment increases in most water bodies as they age and undergo natural eutrophication and succession. We presume that eutrophication in the studied ponds is partly retarded by the construction of wastewater treatment plants (WWTPs) in the catchment upstream of the studied ponds. The luxuriant growth of primary producers due to eutrophication can be regulated by efficient control of nutrient loading [36,37].

The biggest difference in the occurrence of macrophytes is the disappearance of four of the six species of the genus *Potamogeton* in 2021 compared to 2001—*P. filiformis*, *P. lucens*, *P. pectinatus,* and *P. trichoides. P. filiformis* and *P. lucens* are listed as “vulnerable” on the Red List [38], while *P. trichoides* is considered an “endangered“ species. In 2001, *P. trichoides* was only found in one pond (P1) with low abundance. Sand-Jensen et al. [23] studied the distribution of macrophytes in Danish lakes and streams over the last 100 years. They also found that, among other species of *Potamogeton*, *P. filiformis* and *P. lucens* have significantly declined in the lakes. In contrast to our results, species typical of eutrophic waters, such as *P. pectinatus*, remained relatively abundant. Our results cannot be directly compared with those of Sand-Jensen et al. [23] since they studied macrophytes in natural lakes. Wiegleb et al. [39] reported that the decline of some rare species is due to random fluctuations in small populations, which can also be the reason for the disappearance of *P. trichoides* from the studied ponds. Successful species have certain characteristics that enable them to compete in habitats with different types of disturbance. Wiegleb et al. [39] also pointed out that crucial aspects of life history for the representatives of the genus *Potamogeton* are reproduction by turions and other fragments, a long-lived rhizome system with deep roots, phenotypic plasticity, and the potential for successful regeneration from remaining buds.

In Europe, it has been documented several times, for example, for rivers in northwest Germany [39], lakes in England [40], and Danish lakes [23], that species of the genus *Potamogeton*, especially those characteristic of oligotrophic waters, are declining. Johnsen et al. [41] recently introduced declining *Potamogeton* species into restored stream reaches in Denmark. However, they discovered that the lack of success could be due to limited overwintering.

Water bodies are dynamic habitats in which species emerge and decline depending on environmental factors, human disturbance, and success in competition with other macrophytes. In 2021, for instance, we recorded three species, namely *Lemna minor*, *Nasturtium officinale*, and *Polygonum amphibium*, in the surveyed ponds that were not observed in 2001. Our results also revealed that stoneworts occurred in five ponds in 2021, while they were absent in 2001, indicating a lower availability of nitrates in the studied ponds during the second survey, as Lambert and Davy [42] claimed that nitrate reduces the growth of *Chara* species. Another indication of the lower availability of nitrogen could be the reduced expansion of filamentous algae [27], which thrived in eight ponds (P1, P4, P6, P7, P8, P9, P10, and P11) twenty years ago, while in 2021, they were recorded to a lesser extent in four ponds (P4, P7, P8, and P9). As the accelerated algae growth is due to a high nutrient concentration in the water [43], we can conclude that the implementation of two WWTPs in the period between the two surveys influenced their decline. The WWTP with 500 PE was commissioned in 2007, while the reconstructed WWTP with 2000 PE started in 2014. These WWTPs collect most of the municipal wastewater from the villages Bašelj, Srednja Bela, and Zgornja Bela, which improves the quality of the Bela Stream, which supplies most of the water for the studied ponds via the Bela Stream and the branching Vršek Canal. A larger WWTP also improves the quality of the water in the Kokra River, which enriches some of these ponds via the pipeline. The improved quality of the incoming water in terms of lower nutrient concentrations was also confirmed by the lower average value of the Ellenberg nutrient indicator values (EIV_N), which was significantly lower (*p* = 0.001) in 2021 than in 2001 (Figure 4).

The alien invasive species *E. canadensis* also grew in ponds P10 and P11 in 2001 but only in P8 in 2021 and only with very low abundance. The lower abundance of this species could be due to the previously mentioned regulated sewage system in the upstream villages, as the species is typical of eutrophic waters [44], or to the mechanical removal of *E. canadensis* by the manager. In addition, species richness in the above ponds increased from 9 to 19 and 11 to 16, respectively, as the invasive *Elodea* disappeared between the two surveys (Table 1).

### 3.2. Effect of Physical and Chemical Properties on the Distribution of Macrophytes in Ponds in 2021

The depth of the ponds in 2021 ranged from 1 m to 4.5 m, while the Secchi depth, representing transparency, was between 0.8 and 2.9 m. A double Secchi depth indicates the end of the photic zone, down to which net photosynthesis is still possible, which is crucial for the growth and reproduction of submerged macrophytes [45]. In the studied ponds, submerged macrophytes only thrived at depths greater than 1 m, so a relatively low Secchi depth negatively affected the occurrence of submerged macrophytes. The lowest Secchi depth was measured in P3 and P5 (Table 2), where no submerged macrophytes were present. The exceptions were P1 and P9, where an even lower Secchi depth resulted from the shallowest depth of these ponds and corresponded to the depth of these ponds.

The lowest measured water temperature was in pond 9 and the highest in pond 6. Pond 9 is located upstream of the Vršek Stream and provides colder water that warms up later due to increasing total residence time in a series of ponds exposed to the sun. Pond 9 is also densely overgrown with emergent macrophytes that shade the water surface (Figure 5).

The electrical conductivity in the ponds ranges from 364 to 4l7 µS/cm, which is higher than in natural alpine lakes in Slovenia [46]. The low concentration of nitrates and phosphates in the water indicates that the higher conductivity is due to other forms of ions (e.g., carbonate, calcium, potassium, magnesium, sulphate, silicate, and chloride) present in the water due to the predominance of carbonate bedrock in the catchments of all water-supplying watercourses in the area.

The results of the RDA revealed that water temperature and transparency had a statistically significant effect (*p* < 0.05) on the presence and abundance of macrophytes in the ponds (Figure 6). *Myriophyllum spicatum* is positively correlated with transparency, meaning that the clearer the water, the better this species thrives. *Schoenoplectus lacustris* is positively correlated with temperature, while *Sparganium erectum* agg. is negatively correlated. Temperature fluctuations affect the abiotic parameters of the water, e.g., the dissolved oxygen content of the water, and they also influence biotic variables such as plankton biomass [47].

The transparency and temperature of the water affected the presence and abundance of macrophytes. Species such as *M. spicatum*, *Ranunculus trichophyllus*, *Elodea canadensis*, and *P. crispus* grow in greater abundance where water transparency is higher. Although we could not confirm that submerged and floating macrophytes are significantly affected by temperature and nutrients, the research by Wu et al. [48] showed that the submerged species *E. canadensis* and *P. crispus* are significantly affected by these two abiotic factors. Their results showed that the biomass of *E. canadensis* increased directly with increasing temperature. In contrast, the biomass of *P. crispus* decreased indirectly with phosphorus enrichment due to increased competition between primary producers. At higher water temperatures, nutrients are more easily released from sediments [49], especially phosphorous, which facilitates floating and emergent species as well as algal blooms [50] and reduces transparency. These facts are concerning due to global warming and the construction of new reservoirs in agricultural landscapes.

The results of NMDS analyses revealed a unification of macrophyte assemblages within 20 years when the taxa matrix was used (Figure 7). Still, this unification was much milder, as reported by Hassall et al. [30] or Sayer and Greaves [29]. Similar to the result of DCA (Figure 2), we can also see the shift in preference for nutrients when the taxa matrix was replaced by nutrient groups (Figure 8) according to EIV_N, which confirms the important effect of nutrient removal due to construction of WWTPs.

## 4. Materials and Methods

### 4.1. Study Area

The Brdo Estate is located in the Upper Carniola region in the municipality of Kranj, approximately 407 m above sea level. A temperate continental climate of central Slovenia (pre-Alpine climate) is typical for this area [51], where the average annual amount of precipitation is 1500–1600 mm. The Public economic institute Brdo Protocol Services of the Republic of Slovenia manages the Brdo Estate. It covers 478 hectares of controlled land, with many plant and animal species. The estate is considered a natural value of national importance, a special-purpose hunting ground, and a Natura 2000 protected area.

**Figure 9:**
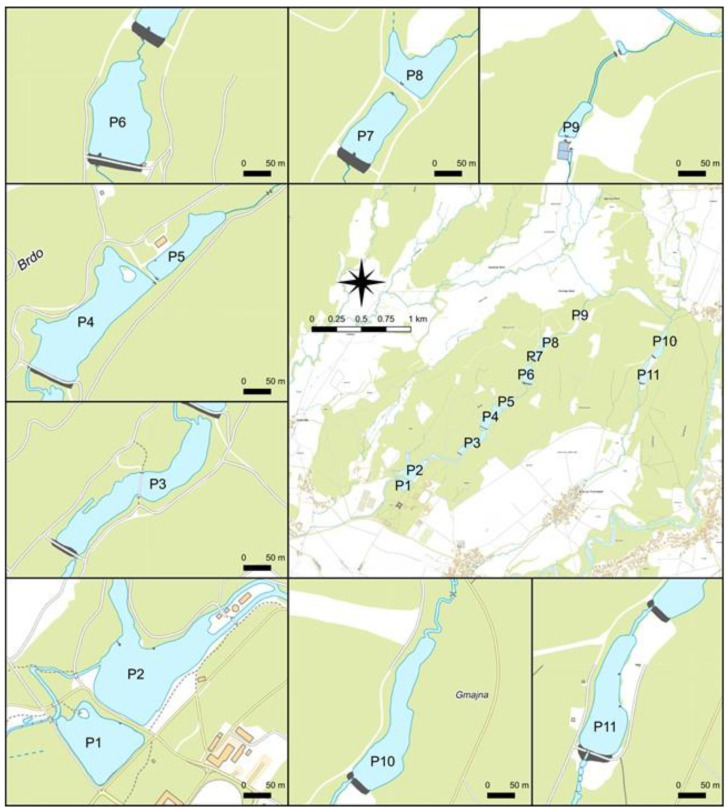
Map of 11 ponds (from P1 to P11) in the studied area (created by M. Holcar). Blue color—ponds; green color—forests; white—agricultural land; black—dams enabling the formation of ponds.

Eleven ponds (P) on the Brdo Estate are fed with water from the Bela Stream. Two of them were created by damming the Bela Stream, whereas nine of them were created by damming the Vršek Stream, which was formed with the bifurcation of the Bela Stream (Figure 9). These dam ponds were formed gradually throughout the estate’s 500-year history; the first (P1) and second (P2) ponds, which are a part of the park near the chateau, are the oldest. Over the centuries, the estate has grown from 66 to almost 500 ha. The park of the Brdo Estate and the ponds are classified among the natural values of national importance. The ponds extend from the castle pond P1 upstream the course of the Vršek Stream to pond P9 and further to the Bela ponds P10 and P11. Water from the Kokra River was piped into ponds P2, P3, P4, P5, and P11 to increase the water flow through these ponds. All ponds are important habitats for many animal and plant species [52].

Various maintenance work has been carried out on some of the ponds in recent years. These included the removal of sediment from P11 in 2010, which was densely overgrown with *E. canadensis*; the installation of a constructed wetland upstream of P9, which helped to reduce nutrient concentrations; and the removal of sediment from the northern part (not surveyed) of P1 in 2020.

In the catchment areas of the water-supplying watercourses (the Bela Stream and the Kokra River) upstream the Brdo Estate, important measures were carried out, such as the construction of a wastewater treatment plant (WWTP) with 4000 PE in 2014 and a WWTP with 500 PE in 2007, which treats the wastewater of most households and farms in the catchment area and thus contributes significantly to the reduction in nutrient concentrations in the water supplying the investigated ponds.

### 4.2. Sampling Design

Our research was performed in 11 ponds with a total surface area of 13.96 ha. A standardized methodology was used to determine the macrophytes in 2001 and 2021. The presence and abundance of macrophytes in the entire littoral zone of each pond was recorded. In addition to the macrophyte survey, basic environmental parameters were also measured.

### 4.3. Macrophyte Survey

The macrophyte survey was carried out from a rowing boat. At each pond, we surveyed the macrophytes of the entire littoral zone. We determined the presence and abundance of macrophytes in each pond on the 5-degree scale [53]. The values of the 5-degree scale were converted into the percentage cover for each taxon, according to Engloner [54]. Species depth occurrence was measured using a depth meter (Speedtech Instruments; Unionville, VA, USA). According to ecological requirements, plants were classified into four groups of ecological types according to Janauer et al. [55], namely submerged macrophytes (SM), floating-leaved macrophytes (FLM), amphiphytes (AM), and emergent macrophytes (EM) or helophytes, and into five groups according to the Ellenberg indicator value (EIV) [56] for nutrient requirements, as presented in Table 3.

According to the Ellenberg indicator value for humidity and nutrients, the average EIV_H per taxon and the average EIV_N per taxon were calculated for each pond based on the composition of the specific plant assemblage.

The Shannon–Wiener diversity index (S-W) was calculated to assess macrophyte diversity.

### 4.4. Physical and Chemical Parameters of Water

The selected physical and chemical water parameters were measured in 2021 using the multiparameter (EUTECH, PCD 650, Singapore). These selected parameters were temperature (°C), pH value, oxygen saturation (%), oxygen content (mg/L), and electrical conductivity (EC) (µS/cm). The water samples were analyzed in the laboratory for PO_4_^3−^ and NO_3_–N content using the HACH Lange (Loveland, CO, USA) LCK (339) and LCK (349) cuvette tests. The absorbance values of the samples were measured with the HACH Lange LT 200 spectrophotometer.

### 4.5. Statistical Analysis

A series of multivariate analyses were performed to reveal the relationships between the structure of the macrophyte community and the ecological requirements in the studied ponds as well as the changes over the last 20 years. First, the detrended correspondence analysis (DCA) was conducted, informing us whether the gradients in the matrix of macrophyte species data were linear or unimodal and which direct gradient analysis we should choose for further analyses. Since the eigenvalue for the first axis was less than 0.4, RDA was selected, as suggested by ter Braak and Verdonschot [57]. DCA was also used to determine the relationships between the macrophyte community structure and the gradients obtained using Ellenberg indicator values passively projected onto the ordination diagram. The influence of physical and chemical parameters on the presence and abundance of macrophytes in the ponds studied in 2021 was determined using multivariate RDA analysis. Data were previously transformed by log transformation (Y′ = log_10_ ((Y + 1). We used forward selection, with 499 permutations per round (Monte Carlo permutation test, pseudo F = 1.7, *p* = 0.004), to find the relative importance of the variables and avoid co-linearity. All analyses were performed using the CANOCO program package for Windows 4.5 (Microcomputer Power: Ithaca, NY, USA) [58].

The differences in Ellenberg indicator values for humidity (EIV_H) and nutrients (EIV_N); the Shannon–Wiener diversity index; the cover of ecological groups of plants such as SM, FLM, AM, and EM in the ponds; and the percentage of plants belonging to groups with EIV_N of 1–3 (N_1-3), 4–5 (N_4-5), 6–7 (N_6-7), and 8 (N_8) between the years 2001 and 2021 were analyzed using the non-parametric Mann–Whitney to determine significant difference between two years.

The differences between the surveys were also examined using NMDS tests with different matrices (such as plant taxa and preferences for nutrient availability) based on Bray–Curtis distance measures (RStudio Software (R version 4.2.3)). We chose this option due to the numerous null values in our data matrix. The data were first standardized (Wisconsin double standardization) and transformed (SQRT transformation). To check if there were statistical differences in species composition and nutrient availability preferences between 2001 and 2021, ANOSIM analysis was performed (RStudio Software (R version 4.2.3)).

## 5. Conclusions

It was found that the succession in the ponds led to an increased number of emergent macrophyte species. On the other hand, eutrophication in the ponds was partly slowed down by the construction of sewage systems in the villages upstream of the watercourses that supply the water for the studied ponds. We conclude that one of the reasons for the lower abundance of submerged macrophytes is the lower transparency caused by fish resuspending the soft sediments. One difference between 2001 and 2021 regarding aquatic plant species abundance is the disappearance of four vulnerable Red List species from the genus *Potamogeton*. Although these ponds are artificial waterbodies created for different purposes, they are important for the conservation of aquatic plant diversity, significantly influencing the ecological balance in the ponds and providing habitats for many other vulnerable species. Proper management of the ponds and their catchment areas is essential for the conservation of aquatic plant diversity as well as the diversity of other aquatic biota.

## Figures and Tables

**Table 1:** Comparison of the presence of macrophyte taxa in 2001 and 2021. (The narrowest line means abundance 1; the thickest line means abundance 5).

	Pond and Year	P1 ′01	P1 ′22	P2 ′01	P2 ′22	P3 ′01	P3 ′22	P4 ′01	P4 ′22	P5 ′01	P5 ′22	P6 ′01	P6 ′22	P7 ′01	P7 ′22	P8 ′01	P8 ′22	P9 ′01	P9 ′22	P10 ′01	P10 ′22	P11 ′01	P11 ′22
	Nr. of SM taxa	4	1	0	2	0	0	2	1	0	0	4	0	3	2	2	5	2	1	1	1	3	4
	Abundance of SM taxa	9	2	0	4	0	0	6	1	0	0	12	0	8	3	8	9	8	1	5	4	8	9
	Plant taxa	14	11	9	23	10	15	12	17	6	16	16	20	15	14	12	14	12	14	11	16	9	19
SM	*Chara sp.*	— — —	— —		— —				—			— — —		— — —	—		—				— — — —		— —
SM	*Elodea canadensis*																—			— — — — —		— — — — —	
SM	*Myriophyllum spicatum*	— —			— —			— — —							— —		— — —						— — — —
SM	*Potamogeton berchtoldi*																					— —	— —
SM	*Potamogeton crispus*											— — —		— — —		— — — —	— —	— — — — —					
SM	*Potamogeton crispus*											— — — —											
SM	*Potamogeton lucens*	— — —						— — —															
SM	*Potamogeton pectinatus*											— —											
SM	*Potamogeton trichoides*	— —																					
SM	*Ranunculus trichophyllus*													— —		— — — —	— —	— — — —	—			— —	—
FLM	*Lemna minor*																—		— —				
FLM	*Nuphar luteum*	— —	— —																				
FLM	*Nymphaea alba*	— — — —	— — — — —																				
AM	*Gratiola officinalis*											— —											
AM	*Mentha aquatica*		—		—	— — —	— —	— — —	— —		— —		— —	— —	—	— —	— —		— —	— — —	— —	— —	— — —
AM	*Myosotis scorpioides*											— —											
AM	*Polygonum amphibium*		—																				
AM	*Veronica beccabunga*																	—	—				
EM	*Alisma plantago-aquatica*				—				—		—	— —	—		—	— —					—		
EM	*Bidens tripartita*												—										
EM	*Caltha palustris*						—	— —	—	—	—	—	—	— —	—	—	— —	— —		—	— —	—	—
EM	*Carex sp.*				— —	— — —	— — —	— —		— — —								—		— — —		— —	—
EM	*Carex acuta*							— — —	— — —		— —		—	— — —	— —	— —	— — —		— — —		— —		
EM	*Carex davalliana*			—	—																		
EM	*Carex elata*	—	—	— — —	— —		— —				— —		—		— —		— —						
EM	*Carex flava agg.*										—	— — —		— —		— —							
EM	*Chaerophyllum hirsutum*				—		—		—		—												
EM	*Eleocharis palustris*										—					—							
EM	*Epilobium palustre*																	—		— —			
EM	*Equisetum palustre*						—		— —		—		— —			—		— —	—				—
EM	*Galium palustre*			— — —	—	— — —	—	— —	—		—	— —	—	—	—	— —	—		—		—		
EM	*Iris pseudacorus*		— —		— —								—							—			
EM	*Juncus effusus*																						—
EM	*Juncus inflexus*		—				—						—		—		— —		— —		—		— — —
EM	*Leersia oryzoides*				—		— —		— —		—		— —		— —		—		— —		— —		— — —
EM	*Lycopus europaeus*		—	— — —	—	— —	— —	— —	— —		—	— —	— —	— —	—	— —	— —	—	— —	—	— —		— — —
EM	*Lysimachia nummularia*			—	—	—			— —	—	—		—										
EM	*Lysimachia vulgaris*				—		—		— —		—		— —		—				—		—		—
EM	*Lythrum salicaria*	— —	—		— —				—				—							—			
EM	*Mentha longifolia*						—					— —		— —				— — —	—	— — —	—	— —	—
EM	*Nasturtium officinale*				—								—								—		—
EM	*Petasites hybridus*				—		—						—										
EM	*Phalaris arundinacea*																						—
EM	*Phragmites australis*					—	— —																
EM	*Rumex hydrolapathum*											—		—									
EM	*Schoenoplectus lacustris*	— —				— — — —		— —	— —			— — —	—	— — —		— —		— — —		— —		— —	
EM	*Scirpus sylvaticus*				—	— —		— —				— — —		— —									
EM	*Scutellaria galericulata*	—		—	—	—	—		—				—	— —							—		
EM	*Solanum dulcamara*	—		— — —	—	— —		—	—	— —			—		—					— —	—		—
EM	*Solidago canadensis*	— —																					
EM	*Sparganium erectum* agg.	—	—	—	—					—	— —							— —	— — — —		—		— — — —
EM	*Typha latifolia*	—		— — —	— —			—		— —	— —	— — —		— —	—			—	— —		—	—	—

**Table 2:** Physical and chemical characteristics of water in ponds (P) in 2021.

Pond	Temperature (°C)	pH	O_2_ Saturation (%)	Dissolved O_2_ (mg/L)	Conductivity (µS/cm)	Nitrate (mg/L)	Secchi Depth (m)	Depth (m)
P1	17.1	7.9	72	6.6	417	2.02	1.0	1.0
P2	17.8	8.3	117	10.5	405	2.02	1.3	3.4
P3	16.8	8.2	111	10.2	400	1.51	1.2	2.9
P4	18.9	8.2	111	9.7	389	1.54	1.4	3.2
P5	18.2	8.0	102	9.1	376	1.73	0.8	1.6
P6	19.9	8.6	119	10.3	364	0.11	1.2	2.8
P7	16.8	8.6	127	11.7	385	0.96	1.3	3.1
P8	16.1	8.7	134	12.4	396	2.66	2.9	4.1
P9	11.5	7.8	81	8.4	388	5.53	1.1	1.1
P10	16.2	7.0	133	12.4	388	1.4	1.65	3.5
P11	15.0	6.1	119	11.3	408	2.59	2.95	4.5

**Table 3:** Groups according to Ellenberg indicator values for nutrients (EIV_N) into which the plant taxa were classified.

EIV (Nutrients)	Nutrients Requirements	Abbreviation
1–3	low	N_1-3
4–5	medium	N_4-5
6–7	high	N_6-7
8	very high	N_8
X	indifferent	N_X

## Data Availability

Data will be available upon a reasonable request.

## Supplementary Materials

The following supporting information can be downloaded at: https://www.mdpi.com/article/10.3390/plants13172439/s1, Figure S1: Coverage with submerged macrophytes for Brdo ponds in 2001 and 2021; Figure S2: Coverage with helophytes for Brdo ponds in 2001 and 2021.
