# Supplementary material for: Aquatic Plants in Ponds at the Brdo Estate (Slovenia) Show Changes in 20 Years
Source: Plants (Basel). 2024 Aug 31;13(17):2439. doi: 10.3390/plants13172439 (PMC11397002; doi:10.3390/plants13172439)
Supplement: Supplementary file 1 [file plants-13-02439-s001.zip › plants-3144190-supplementary.pdf]

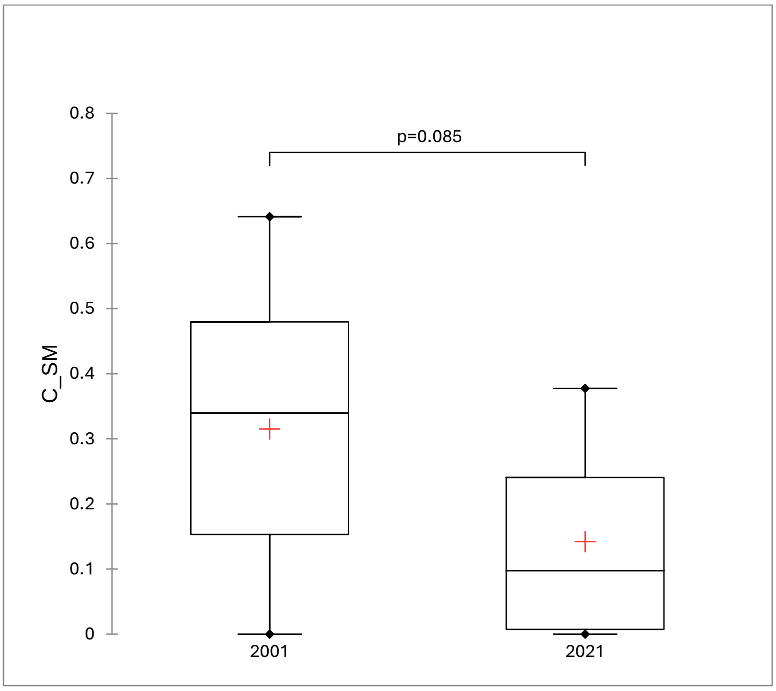


Supplement 1 Fig. S1: Coverage with submerged macrophytes for Brdo ponds in 2001 and 2021.

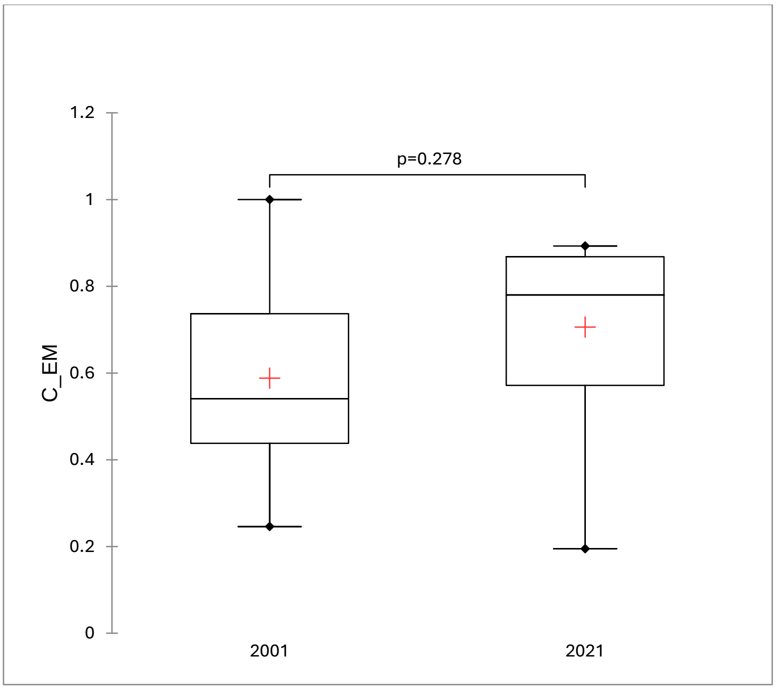


Supplement 1 Fig. S2: Coverage with helophytes for Brdo ponds in 2001 and 2021.
